# Single-dose nonavalent HPV vaccine: Need of the hour

**DOI:** 10.3126/nje.v10i2.28962

**Published:** 2020-06-30

**Authors:** Manidip Pal, Soma Bandyopadhyay

**Affiliations:** 1 Professor & HOD, Obstetrics & Gynecology, College of Medicine & JNM Hospital, WBUHS, Kalyani, Nadia, West Bengal, India; 2 Professor & HOD, Obstetrics & Gynecology, Katihar Medical College, Karimbagh, Katihar, Bihar, India

**Keywords:** HPV, nonavalent, quadrivalent, single-dose, vaccine

## Abstract

Human Papilloma Virus (HPV) vaccination of the preadolescent (9-14 years) females is the potential solution to eradicate carcinoma cervix. Nonavalent vaccine provides wider coverage than the quadrivalent vaccine. On long-term follow-up, even after single-dose HPV vaccination, the antibody titer remains good. Herd immunity can also be achieved by HPV vaccination. Hence, mass single-dose nonavalent HPV vaccination for sexually naive preadolescent girls can provide almost 100% protections and a cost-effective approach for the developing countries.

## Introduction

Cervical cancer is a preventable disease and whole world is now focusing on prevention of carcinoma cervix. Cervical intraepithelial neoplasia (CIN) is the forerunner of cervical cancer. HPV infection is invariably associated with CIN. In carcinoma cervix, HPV type 16 and 18 are responsible for about 70% cases and HPV type 31, 33 and 45 are responsible for another 13% cases. Hence, HPV vaccination is the key to eradicate carcinoma cervix. There are various HPV vaccines been invented so far including bivalent targeting HPV (type 16 and 18); quadrivalent targeting HPV (type 6,11,16,18); and nonavalent targeting HPV (type 6,11,16,18,31,33,45,52,58). Human papillomavirus 9-valent vaccine, recombinant; 9vHPV (Gardasil 9) was approved by FDA in 2014. Since the end of 2016, only 9-valent HPV vaccine is available in the United States [1].

### Hypothesis of single dose HPV vaccination

In the multi-centric Indian clinical trial (NCT00923702) quadrivalent HPV vaccine was given and the participants were ended up in 4 groups – 1) 3 dose group: girls vaccinated on days 1, 60 and 180 or later, 2) 2 dose group: girls vaccinated on days 1 and 180 or later, 3) 2 dose group: girls vaccinated on days 1 and 60 by default and 4) Single dose: by default because administration of vaccine was stopped in April 2010 by Govt of India [2]. Interestingly, same efficacy of single dose vs 2 or 3 dose vaccine was reported. Immunogenicity following 2 doses of HPV vaccination was non-inferior to 3 doses vaccination, and single dose recipients showed a robust and sustained immune response against HPV 16 and 18, albeit inferior to that seen after 2 and 3 doses. Antibody levels were stable over a 4-year period [2,3]. The short term protection by single dose HPV vaccine against persistent infection with HPV 16, 18, 6, and 11 is similar to that by 2 or 3 doses of vaccine and merits further assessment [4]. US National Cancer Institute Costa Rica trial revealed that though the antibody levels after single dose bivalent HPV vaccine was lower than the levels after 3 doses, but it was 9-times higher than levels elicited by natural infection. Importantly, antibody levels remained essentially constant over 7 years [5]. Forty years after initiating routine vaccination and depending on assumptions of vaccine waning, single-dose HPV vaccination with equivalent coverage (70%) averted 15–16% of carcinoma cervix cases vs 21% with 2-dose vaccination, but required only half economic investment [6]. Single-Dose HPV Vaccine Evaluation Consortium (2018-2021), a group of nine leading independent research institutions are working together to collect and synthesize existing evidence and evaluate new data on the potential for single-dose HPV vaccination and in their report on January 15, 2020 they commented that evidence proving that single dose HPV vaccination may be enough to elicit protective immune response against HPV infection [7].

### Herd immunity

HPV vaccine also provides herd immunity. While comparing data of 2003–2006 to 2011-2014 it was found that, in sexually active women of 14-24 years the prevalence of HPV is reduced by 89% in vaccinated women (4vHPV) and 34% in unvaccinated women [8]. After 10 years of vaccination, the HPV detection rate decreased from 35% to 6.7% (80.9% decline) among vaccinated women; whereas HPV detection rate decreased from 32.4% to 19.4% (40% decline) among unvaccinated [9].

### Cost-effectiveness

Australia, United Kingdom and Denmark have achieved high coverage of their population by including HPV vaccination in their national immunization programme [1]. In India, states of Punjab and Sikkim had also started vaccination among adolescent girls. Negotiations with the manufactures can possibly reduce the cost per dose for a huge market like India to the extent of 1$, a price that is considered affordable for immunization program in a developing country [10]. Single dose HPV vaccination resulted in cost-savings compared to no vaccination and could be cost-effective compared to two-dose vaccination [6]. National manufacturing unit as well as private manufacturing companies are producing or going to produce 4 valent HPV vaccine whereas nonavalent HPV vaccination is the pathway to eradicate carcinoma cervix. Therefore, it would be ideal to invest money in production of nonavalent HPV vaccine which is the need of the hour.

## Conclusion

Considering all these facts, it would be prudent to initiate the process of including single dose nonavalent HPV vaccination for preadolescent sexually naive girls in the national immunization schedule.

## References

[1] Markowitz LE Gee J Chesson H Stokley S. Ten years of Human Papillomavirus vaccination in the United States. Acad Pediatr. 2018;18(2S):S3-S10. 10.1016/j.acap.2017.09.014 PMid: 29502635 PMC11331487

[2] Sankaranarayanan R Joshi S Muwonge R Can a single dose of human papillomavirus (HPV) vaccine prevent cervical cancer? Early findings from an Indian study. Vaccine. 2018;36(32PtA):4783-4791. 10.1016/j.vaccine.2018.02.087 PMid: 29551226

[3] Sankaranarayanan R Basu P Kaur P Current status of human papillomavirus vaccination in India's cervical cancer prevention efforts. Lancet Oncol. 2019;20(11):e637-e644. 10.1016/S1470-2045(19)30531-5 31674322

[4] Sankaranarayanan R Prabhu PR Pawlita M Immunogenicity and HPV infection after one, two, and three doses of quadrivalent HPV vaccine in girls in India: a multicentre prospective cohort study. Lancet Oncol. 2016;17(1):67-77. 10.1016/S1470-2045(15)00414-3 26652797 PMC5357737

[5] Kreimer AR Herrero R Sampson JN Evidence for single-dose protection by the bivalent HPV vaccine-Review of the Costa Rica HPV vaccine trial and future research studies. Vaccine.2018;36(32PtA):4774-4782. 10.1016/j.vaccine.2017.12.078 PMid: PMCid: 29366703 PMC6054558

[6] Burger EA Campos NG Sy S Regan C Kim JJ Health and economic benefits of single-dose HPV vaccination in a Gavi-eligible country. Vaccine 2018; 36:4823-4829. 10.1016/j.vaccine.2018.04.061 PMid: PMCid: 29807710 PMC6066173

[7] Newton T. (2020). Will a single dose of HPV vaccine prevent cervical cancer? [online].

[8] Oliver SE Unger ER Lewis R Prevalence of human papilloma virus among females after vaccine introduction - National Health and Nutrition Examination Survey, United States, 2003-2014. Infect Dis. 2017;216(5):594-603. 10.1093/infdis/jix244 PMid: PMCid: 28931217 PMC5740482

[9] Spinner C Ding L Bernstein DI Brown DR Human papillomavirus vaccine effectiveness and herd protection in young women. Pediatrics. 2019;143(2):e20181902. 10.1542/peds.2018-1902 PMid: PMCid: 30670582 PMC6361347

[10] Basu P Banerjee D Singh P Bhattacharya C Biswas J. Efficacy and safety of human papillomavirus vaccine for primary prevention of cervical cancer: A review of evidence from phase III trials and national programs. South Asian J Cancer 2013; 2(4):187-192. 10.4103/2278-330X.119877 PMid: PMCid: 24455618 PMC3889021

